# Antioxidant Responses of Ragweed Leaf Beetle *Ophraella communa* (Coleoptera: Chrysomelidae) Exposed to Thermal Stress

**DOI:** 10.3389/fphys.2018.00808

**Published:** 2018-07-06

**Authors:** Hongsong Chen, Ghulam Sarwar Solangi, Jianying Guo, Fanghao Wan, Zhongshi Zhou

**Affiliations:** ^1^State Key Laboratory for Biology of Plant Diseases and Insect Pests, Institute of Plant Protection, Chinese Academy of Agricultural Sciences, Beijing, China; ^2^Guangxi Key Laboratory for Biology of Crop Diseases and Insect Pests, Institute of Plant Protection, Guangxi Academy of Agricultural Sciences, Nanning, China; ^3^Department of Entomology, Sindh Agriculture University Subcampus, Umerkot, Pakistan

**Keywords:** *Ophraella communa*, thermal stress, total protein, antioxidant enzymes activity, biological control

## Abstract

*Ophraella* *communa* LeSage is an effective biological control agent of common ragweed, *Ambrosia artemisiifolia* L., which competes with crops and causes allergic rhinitis and asthma. However, thermal stress negatively affects the developmental fitness and body size of this beetle. High temperatures cause a variety of physiological stress responses in insects, which can cause oxidative damage. We investigated the total protein content and activity of antioxidant enzymes including superoxide dismutase (SOD), catalase (CAT), and peroxidases (PODs) in *O*. *communa* adults when its different developmental stages were exposed to high temperatures (40, 42, and 44°C) for 3 h each day for 3, 5, 5, and 5 days, respectively (by stage), and a whole generation to high temperatures (40, 42, and 44°C) for 3 h each day. A control group was reared at 28 ± 2°C. Under short-term daily phasic high-temperature stress, total protein contents were close to the control as a whole; overall, SOD activities increased significantly, CAT activities were closer to or even higher than the control, POD activities increased at 40°C, decreased at 42 or 44°C; stage-specific response was also observed. Under long-term daily phasic high-temperature stress, total protein content increased significantly at 44°C, SOD activities increased at higher temperatures, decreased at 44°C; CAT activities of females increased at ≤42°C, and decreased at 44°C, CAT activities of males decreased significantly; POD activities of females increased at 40°C, decreased at ≥42°C, POD activities of males decreased at 44°C; and antioxidant enzymes activities in females were significantly higher than those in males. Antioxidative enzymes protect *O*. *communa* from oxidative damage caused by thermal stress.

## Introduction

Temperature is one of the most important environmental factors affecting life history, behavioral and physiological traits, population structure, and community composition in insects (Zhang et al., 2015b; Esperk et al., 2016). Insects have an optimal temperature range to which their biological functions are best adapted; over this range, insects might suffer physiological costs and fitness decrease (Jerbi-Elayed et al., 2015). Climate change has led to an increase in the frequency, intensity, and length of extreme high temperatures around the globe and this trend is expected to continue (Ma et al., 2018). In many parts of China, summer daily maximum temperatures in the field often exceed 40°C for several hours, and the number of such hot days has also increased in the last few years (Zhang et al., 2015b). Small insect herbivores often have short life cycles leading to overlapping generations, so any life stage may experience heat stress and the effects of heat stress also depend on heat stress (Zhao et al., 2017).

Thermal stress can result in oxidative damage and oxidative stress (Lopez-Martinez et al., 2008; Zhang et al., 2014). Oxidative stress refers to elevated intracellular levels of reactive oxygen species (ROS) that cause damage to lipids, proteins, and DNA (Schieber and Chandel, 2014). To prevent damage from ROS, insects have developed antioxidant defense mechanisms and these systems have both enzymatic and non-enzymatic components (Felton and Summers, 1995); antioxidant enzymes are key components in the regulation of intracellular ROS balance (Wang et al., 2012; Jia et al., 2014; Li et al., 2017). Major antioxidative enzymes in insects include superoxide dismutase (SOD), catalase (CAT), and peroxidases (POD) which are reported to be involved in insect defense systems (Zhang et al., 2015a; Li et al., 2017). SOD converts superoxide anion (O_2_^-^) into oxygen (O_2_) and hydrogen peroxide (H_2_O_2_), and CAT and POD break down hydrogen peroxide (H_2_O_2_) into oxygen and water (Covarrubias et al., 2008; Li et al., 2017), which protect insects from oxidative damage.

*Ophraella communa* LeSage (Coleoptera: Chrysomelidae) is the best biological control agent of common ragweed, *Ambrosia artemisiifolia* L. (Asterales: Asteraceae) (Zhou et al., 2017). The larvae and adults of this leaf beetle feed on *A. artemisiifolia* leaves, destroying plants when its adults and larvae reach a high density, thus it performs a good control efficacy on *A. artemisiifolia* (Zhou et al., 2017). *A. artemisiifolia* is one of the most noxious weeds in agriculture around the world (Wan and Wang, 1988), due to its significantly negative effects on agricultural production, allergic rhinitis and asthma to human and impacts on biodiversity (Zhou et al., 2017). Research on *O. communa* has provided important insights into temperature. At constant temperature in the laboratory, the optimum developmental temperature for *O. communa* ranges from 25 to 28°C, and temperatures not far beyond this range are harmful (Zhou et al., 2010); at ≥36°C, all first-instar larvae are dead within 24 h, and the survival of other instars and female fecundity decreases significantly (Zhou et al., 2010). Under brief heat stress, the pre-adult development and survival, adult survival, longevity, and fecundity of *O. communa* were all adversely affected by 2 h at ≥35°C (Zhou et al., 2011) or 3 h at ≥40°C (Chen et al. unpublished data), the body size of *O. communa* adults is also adversely affected after exposure of immature stages to 3 h at ≥40°C (Chen et al., 2014). However, the over 50% survival rates of eggs, pupae, and adults of *O. communa* exposure to 44°C for 3 h (Chen et al. unpublished data), the effective control of *O. communa* and *Epiblema strenuana* on *A. artemisiifolia* in the field in summer (Zhou et al., 2014) both indicate that *O. communa* has a large thermal tolerance plasticity. To date, the response of antioxidant enzymes in *O. communa* exposed to thermal stress has not been reported. The aim of the present study was to determine the effects of short-term and long-term phasic high-temperature exposure on the anti-oxidant enzymes activities of *O*. *communa* and identify the physiological repair responses to hot summer in the field.

## Materials and Methods

### Host Plants

*Ambrosia artemisiifolia* seeds were collected from more than 10,000 plants from a field (28°56′26^′′^N, 113°14’38^′′^E) near the town of Dajing, in Miluo county, Yueyang city, Hunan province, China, during late October 2010. The seeds were then stored at 4°C. Adequately stored seeds were sown in a greenhouse at 28 ± 2°C under natural light at the Institute of Plant Protection, Hunan Academy of Agricultural Sciences (25°21′18^′′^N, 114°33′40^′′^E), Changsha, Hunan province, China, in late March 2011. When seedlings reached a height of approximately 15 cm, some of them were used in *O. communa* adult heat treatments. Apical buds of the remaining seedlings were removed to prevent apical dominance, and the seedlings were transplanted into pots (21 cm × 17 cm) containing soil with one seedling per pot, a total of 1,000 common ragweed seedlings were prepared. All the plants were watered every day and fertilizer was applied (N:P:K = 13:7:15) twice per month to maintain growth (Zhou et al., 2010). When the plants were approximately 40 cm high, approximately 400 of the potted plants were used for heat treatments of eggs, larvae, and pupae.

### Insect Culture

More than 1,000 *O. communa* adults were collected from *A. artemisiifolia* plants in the same place for the plants in the previous subsection on June 24, 2011. Colonies of the beetle were maintained on *A. artemisiifolia* plants under natural light in the same greenhouse for the seedlings in the previous subsection.

Twelve pairs of *O. communa* adults were randomly collected from the rearing colony, and each individual was placed with the aid of a fine brush (size 0) onto a fresh common ragweed plant in a pot, which was then covered with nylon gauze (40 mesh size). After allowing two days for oviposition, the adult beetles were removed, the plants with newly eggs were placed in the greenhouse for normal growth until lots of the needed life stage emergence (such as eggs ≤ 12-h old, first-instar larvae ≤ 24-h old, pupae ≤ 24-h old, adults ≤ 12-h old).

### Heat Treatment Intensities and Durations

The durations and intensities of our heat stress treatments were based on the duration and intensity of the highest temperatures in summer, which in central China is usually a few hours on any 1 day (maximum temperature of 44°C for approximately 3 h per day; Chen et al., 2014). Therefore, 40, 42, and 44°C for 3 h per day were selected, the treatment of 28°C was considered as control (Zhou et al., 2010). The exposure periods continue for 3–5 (one developmental stage) or over 20 (one generation) days based on the developmental periods of different developmental stages of *O. communa* (4.0 days for the egg, 7.6 days for the larva, and 6.0 days for the pupa) earlier recorded at a constant high temperature (32°C) in laboratory (Zhou et al., 2010) and the number of hot days (daily maximum temperature ≥ 40°C) at present and in the near future in China (Zhang et al., 2015b; Ma et al., 2018). The high-temperature exposures for each treatment were performed separately in environmental chambers (PRX-450D, Ningbo Haishu Safe Experimental Equipment Co., Ltd., Zhejiang, China) at 28 (control), 40, 42, or 44 ± 1°C, with a relative humidity of 70 ± 5%, a photoperiod of 14:10 (L:D) h (Zhou et al., 2010), and a light intensity of 12,000 lx for 3 h daily. Each treatment was repeated three times.

### Short-Term Phasic Thermal Stress on Eggs, Larvae, Pupae, and Adults of *O. communa*

The experiments were started in early July 2011. One hundred eggs ≤ 12-h old, 100 first-instar larvae ≤ 24-h old, and 50 pupae ≤ 24-h old were separately retained on three potted plants in the greenhouse. Ten ragweed plants were randomly selected for each developmental stage, and they were then exposed to high temperatures for 3 h daily for 3, 5, and 5 consecutive days for eggs, larvae, and pupae in environmental chambers, respectively (by stage), after which the infested potted plants were kept in the greenhouse. A total of 120 ragweed plants were used.

Following high-temperature stress, treated pupae were collected by detaching the leaves they were on and placing the individual leaves into open transparent plastic boxes (19 cm × 12 cm × 6 cm) in an unsealed cuvette plastic tube covered with nylon gauze (60 mesh size) in the laboratory at 28 ± 2°C and 70 ± 5% relative humidity, where pupae were checked daily for adult emergence. The treated eggs and larvae were kept in the greenhouse until they reached the pupal stage. The process for these pupae was the same as that for the treated pupae following high-temperature stress. The sex of each newly emerged adult was determined with the help of stereo microscope. These adults were kept in the laboratory at 28 ± 2°C with relative humidity of 70 ± 5% for 5 days.

Newly emerged adults ≤ 12-h old (45 pairs) in the greenhouse culture were randomly selected for phasic high-temperature exposures. Fifteen pairs were placed on each of three fresh common ragweed seedlings (15 cm height) potted in a plastic box (19 cm × 12 cm × 6 cm) with a hole (15 cm × 4 cm) and covered with nylon gauze (60 mesh size), and this constituted one replicate. Ragweed seedlings containing these adults were exposed to high temperatures in environmental chambers for 3 h daily for 5 consecutive days, after which the infested potted plants were kept in a greenhouse, and a total of 36 ragweed plants were used.

### Long-Term Phasic Thermal Stress on *O. communa*

The experiments were also started in early July 2011. Approximately 1,000 eggs ≤ 12-h old in the greenhouse were retained on one potted plant. Ten potted plants were selected for each treatment temperature, and they were then exposed to the high-temperature treatments in environmental chambers for 3 h daily until the emergence of the adult. After which the infested potted plants were kept in a greenhouse, and a total of 40 ragweed plants were used. Fifteen pairs of newly emerged adults ≤ 12-h old were placed on three fresh common ragweed seedlings (15 cm height) potted in a plastic box (19 cm × 12 cm × 6 cm) with a hole (15 cm × 4 cm) and covered with nylon gauze (60 mesh size), and this constituted one replicate. The *O*. *communa* adults on ragweed seedlings were then exposed to high temperatures in the environmental chambers for 3 h daily for 5 consecutive days, after which the infested potted plants were kept in a greenhouse, and a total of 36 ragweed plants were used.

### Total Protein Content and Enzymes Activity Assays of *O. communa*

After exposure to high temperatures, the total protein content and antioxidant enzyme activity in subsequent adults were determined. The protein extraction protocols were carried out according to a total protein quantitative assay (A045-2, Nanjing Jiancheng Bioengineering Institute, Nanjing, China). Protein concentrations were determined according to the Bradford () method with bovine serum albumin as the standard.

The activities of SOD, CAT, and POD were examined using commercially available assay kits (A001-1-1, A007-1-1, A084-1, Nanjing Jiancheng Bioengineering Institute, Nanjing, China) following the manufacturer’s protocols.

Superoxide dismutase activity was measured spectrophotometrically at 550 nm by xanthine and xanthine oxidase systems. One unit of SOD activity was defined as the amount of enzyme required to cause 50% inhibition of the xanthine oxidase system reaction in 1 ml enzyme extract with 1 mg protein (U mg^-1^ protein). CAT activity was determined spectrophotometrically at 405 nm by measuring the decrease of H_2_O_2_ due to hydrogen peroxide decomposition. One unit of CAT activity was defined as the amount that decomposes 1 μmol of H_2_O_2_ per second per mg protein (U mg^-1^ protein). POD activity was determined at 420 nm by catalyzing the oxidation of a substrate in the presence of H_2_O_2_. One unit of POD activity was defined as the amount that catalyzes 1 μg substrate per minute per mg protein (U mg^-1^ protein; Jia et al., 2011).

### Statistical Analyses

All data were analyzed using SPSS 21.0 (SPSS Inc., Chicago, IL, United States). Means were separated using Tukey’s honestly significant difference (HSD) test (one-way ANOVA) when significant differences were found at *P* < 0.05 and were denoted as the means ± SE (standard error of the mean).

## Results

### Total Protein Content

First, in order to assay the antioxidant enzymes activities after exposure of different developmental stages or whole generation to phasic high temperatures, we evaluated the total protein content in subsequent adults. Total protein contents in *O. communa* females were significantly affected by the previous exposure of larvae (*F*_3,8_ = 52.31, *P* < 0.0001), pupae (*F*_3,8_ = 85.78, *P* < 0.0001), and adults (*F*_3,8_ = 434.51, *P* = 0.0007) to phasic high temperatures, except for eggs (*F*_3,8_ = 3.83, *P* = 0.0571); the total protein contents were stage-specific when different developmental stages were exposed to any phasic high temperature compared to the control (**Table 1**). Total protein contents in *O. communa* males were also significantly affected by the previous exposure of eggs (*F*_3,8_ = 53.87, *P* < 0.0001), larvae (*F*_3,8_ = 48.83, *P* < 0.0001), pupae (*F*_3,8_ = 5.85, *P* = 0.0205), and adults (*F*_3,8_ = 32.49, *P* = 0.0001) to phasic high temperatures; the total protein contents were stage-specific when different developmental stages were exposed to 40 or 42°C for 3 h compared to the control and 44°C (**Table 2**).

**Table 1:** Mean (±SE) protein content of *O. communa* female adults when eggs, larvae, pupae, and adults were exposed for 3 h each day for 3, 5, 5, and 5 days, respectively (by stage), to 28 (control), 40, 42, and 44°C.

Temperature (°C)	Protein content (mg⋅L^-1^)			
	Eggs	Larvae	Pupae	Adults
28	3.3 ± 0.2a, A	3.5 ± 0.1a, A	3.6 ± 0.1b, A	3.5 ± 0.0b, A
40	2.8 ± 0.1b, B	2.0 ± 0.1d, C	5.3 ± 0.2a, A	1.7 ± 0.1c, C
42	3.3 ± 0.2a, B	3.0 ± 0.1b, B	2.3 ± 0.1d, C	4.2 ± 0.1a, A
44	3.4 ± 0.1a, A	3.5 ± 0.0a, A	2.8 ± 0.2c, B	3.5 ± 0.1b, A

*Values within the same column followed by different lowercase and uppercase letters within the same row are significantly different (Tukey’s HSD test, P < 0.05).*

**Table 2:** Mean (±SE) protein content of *O. communa* male adults when eggs, larvae, pupae, and adults were exposed for 3 h each day for 3, 5, 5, and 5 days, respectively (by stage), to 28 (control), 40, 42, and 44°C.

Temperature (°C)	Protein content (mg⋅L^-1^)			
	Eggs	Larvae	Pupae	Adults
28	3.3 ± 0.2a, B	3.4 ± 0.1a, AB	3.8 ± 0.2ab, A	3.2 ± 0.1a, B
40	3.4 ± 0.1a, A	3.4 ± 0.1a, A	3.3 ± 0.1bc, A	3.2 ± 0.1a, A
42	1.3 ± 0.1c, D	3.5 ± 0.1a, B	4.0 ± 0.2a, A	2.1 ± 0.1b, C
44	2.7 ± 0.2b, B	2.0 ± 0.1b, C	3.2 ± 0.1c, A	3.4 ± 0.1a, A

*Values within the same column followed by different lowercase and uppercase letters within the same row are significantly different (Tukey’s HSD test, P < 0.05).*

**FIGURE 1:**
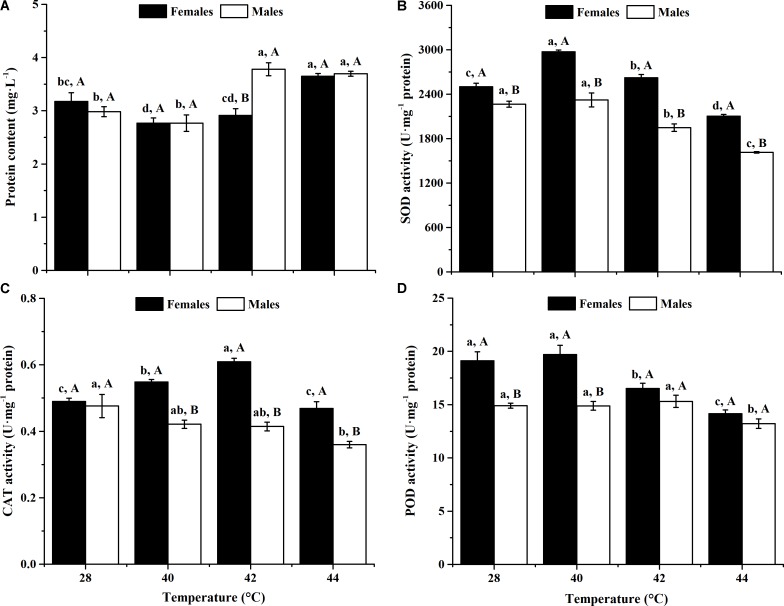
Effect of long-term phasic thermal stress (12-h-old eggs to 5-days-old adults) on protein content and antioxidant enzyme activities of *O*. *communa* adults. The temperature 28°C served as a control. Each value represents the mean (±SE) in adult females (black bars) and males (white bars). Different lowercase letters indicate significant differences among heat treatments for the same gender. Different uppercase letters indicate significant differences between males and females at the same temperature (Tukey’s HSD test, *P* < 0.05). **(A)** Protein content. **(B)** Superoxide dismutase (SOD) activity. **(C)** Catalase (CAT) activity. **(D)** Peroxidase (POD) activity.

After long-term phasic thermal stress, the total protein contents of *O. communa* female (*F*_3,8_ = 10.83, *P* = 0.0007) and male (*F*_3,8_ = 20.50, *P* = 0.0004) adults were significantly affected (**Figure 1A**). Total protein content of female adults significantly increased when exposed to 44°C, and male adults showed significantly increased content at 42 and 44°C, compared with the controls. No significant difference was observed in female adults between the 42°C and control conditions, or in male adults between the 40°C and control conditions (**Figure 1A**).

Taken together, these results suggest total protein contents in subsequent adults were significantly affected by the previous exposure of different developmental stages or whole generation to brief high temperatures.

### Antioxidative Enzymes Activity

Next, we measured the antioxidant enzymes activities of *O. communa* adults after exposure of different developmental stages or whole generation to short- and long-term phasic high temperatures. In total, SOD activities of *O. communa* females increased after exposure of different developmental stages to phasic high temperatures (*P* < 0.05) compared to the control; the SOD activities were also stage-specific when different developmental stages were exposed to any phasic high temperature (*P* < 0.05) compared to the control (**Table 3**). The SOD activities of *O. communa* males also increased significantly by the previous exposure of eggs (*F*_3,8_ = 33.2, *P* < 0.0001), larvae (*F*_3,8_ = 240.02, *P* < 0.0001), and adults (*F*_3,8_ = 23.07, *P* = 0.0003) to phasic high temperatures, but the result for pupae is opposite (*F*_3,8_ = 7.91, *P* = 0.0089); the stage-specific responses were also observed when different developmental stages were exposed to any phasic high temperature compared to the control (**Table 4**).

**Table 3:** Mean (±SE) SOD activity of *O. communa* female adults when eggs, larvae, pupae, and adults were exposed for 3 h each day for 3, 5, 5, and 5 days, respectively (by stage), to 28 (control), 40, 42, and 44°C.

Temperature (°C)	SOD activity (U mg^-1^ protein)			
	Eggs	Larvae	Pupae	Adults
28	2549 ± 68b, A	2493 ± 97b, A	2547 ± 48b, A	2495 ± 55b, A
40	2709 ± 9ab, BC	2814 ± 31a, B	2675 ± 45b, C	4385 ± 42a, A
42	2725 ± 44a, B	2629 ± 41ab, B	3329 ± 41a, A	2087 ± 35c, C
44	2271 ± 66c, B	2530 ± 37b, A	2643 ± 34b, A	2170 ± 53c, B

*Values within the same column followed by different lowercase and uppercase letters within the same row are significantly different (Tukey’s HSD test, P < 0.05).*

**Table 4:** Mean (±SE) SOD activity of *O. communa* male adults when eggs, larvae, pupae, and adults were exposed for 3 h each day for 3, 5, 5, and 5 days, respectively (by stage), to 28 (control), 40, 42, and 44°C.

Temperature (°C)	SOD activity (U mg^-1^ protein)			
	Eggs	Larvae	Pupae	Adults
28	2354 ± 64b, A	2288 ± 69c, A	2354 ± 59a, A	2256 ± 70c, A
40	3010 ± 95a, A	2456 ± 46b, B	2144 ± 49ab, C	2874 ± 46a, A
42	3160 ± 74a, B	3750 ± 27a, A	1982 ± 106b, D	2270 ± 84c, C
44	2464 ± 22b, A	2043 ± 46d, B	1921 ± 44b, C	2537 ± 24b, A

*Values within the same column followed by different lowercase and uppercase letters within the same row are significantly different (Tukey’s HSD test, P < 0.05).*

After long-term phasic thermal stress, the SOD activities of both *O. communa* female and male adults increased at higher temperatures (females at 40 or 42°C, males at 40°C), decreased at the highest temperature (**Figure 1B**). SOD activities of female adults were significantly higher than those of males both at high temperatures and 28°C (**Figure 1B**).

Overall, the CAT activities in *O. communa* adults were closer to or even higher than the control after exposure of different developmental stages to phasic high temperatures; the stage-specific responses were also observed when different developmental stages were exposed to any phasic high temperature compared to the control (**Tables 5, 6**).

**Table 5:** Mean (±SE) CAT activity of *O. communa* female adults when eggs, larvae, pupae, and adults were exposed for 3 h each day for 3, 5, 5, and 5 days, respectively (by stage), to 28 (control), 40, 42, and 44°C.

Temperature (°C)	CAT activity (U mg^-1^ protein)			
	Eggs	Larvae	Pupae	Adults
28	0.50 ± 0.02b, A	0.48 ± 0.02b, A	0.51 ± 0.02b, A	0.48 ± 0.02b, A
40	0.64 ± 0.02a, C	0.81 ± 0.02a, B	0.32 ± 0.01c, D	1.08 ± 0.08a, A
42	0.45 ± 0.01b, B	0.56 ± 0.04b, A	0.62 ± 0.01a, A	0.47 ± 0.02b, B
44	0.47 ± 0.01b, B	0.55 ± 0.03b, A	0.35 ± 0.01c, C	0.49 ± 0.01b, B

*Values within the same column followed by different lowercase and uppercase letters within the same row are significantly different (Tukey’s HSD test, P < 0.05).*

**Table 6:** Mean (±SE) CAT activity of *O. communa* male adults when eggs, larvae, pupae, and adults were exposed for 3 h each day for 3, 5, 5, and 5 days, respectively (by stage), to 28 (control), 40, 42, and 44°C.

Temperature (°C)	CAT activity (U mg^-1^ protein)			
	Eggs	Larvae	Pupae	Adults
28	0.41 ± 0.02c, A	0.44 ± 0.04b, A	0.40 ± 0.02a, A	0.41 ± 0.01c, A
40	1.04 ± 0.05a, A	0.40 ± 0.01bc, C	0.41 ± 0.01a, C	0.58 ± 0.03a, B
42	0.47 ± 0.01bc, C	0.80 ± 0.01a, A	0.31 ± 0.02b, D	0.52 ± 0.01b, B
44	0.54 ± 0.01b, A	0.35 ± 0.01c, C	0.40 ± 0.01a, B	0.29 ± 0.01d, D

*Values within the same column followed by different lowercase and uppercase letters within the same row are significantly different (Tukey’s HSD test, P < 0.05).*

After long-term phasic thermal stress, the CAT activities of *O. communa* female adults increased at 40 and 42°C, decreased at 44°C (*F*_3,8_ = 34.45, *P* < 0.0001); the CAT activities of male adults decreased significantly at high temperatures compared to the control (*F*_3,8_ = 5.52, *P* = 0.0238); higher CAT activities were observed in female adults than those of males both at high temperatures and control (**Figure 1C**).

Overall, the POD activities of both *O. communa* female and male adults increased at 40°C, decreased at 42 or 44°C; the stage-specific responses were also observed when different developmental stages were exposed to any phasic high temperature compared to the control (**Tables 7, 8**).

**Table 7:** Mean (±SE) POD activity of *O. communa* female adults when eggs, larvae, pupae, and adults were exposed for 3 h each day for 3, 5, 5, and 5 days, respectively (by stage), to 28 (control), 40, 42, and 44°C.

Temperature (°C)	POD activity (U mg^-1^ protein)			
	Eggs	Larvae	Pupae	Adults
28	18.20 ± 2.11a, A	19.64 ± 0.40b, A	18.81 ± 0.94a, A	18.78 ± 0.84a, A
40	20.86 ± 0.89a, AB	22.56 ± 1.04a, A	19.21 ± 0.61a, B	19.57 ± 0.89a, B
42	12.41 ± 0.61b, BC	13.35 ± 0.56c, B	20.26 ± 0.61a, A	10.57 ± 0.71b, C
44	12.27 ± 0.56b, B	9.89 ± 0.23d, C	15.43 ± 0.48b, A	10.70 ± 0.83b, BC

*Values within the same column followed by different lowercase and uppercase letters within the same row are significantly different (Tukey’s HSD test, P < 0.05).*

**Table 8:** Mean ( ± SE) POD activity of *O. communa* male adults when eggs, larvae, pupae, and adults were exposed for 3 h each day for 3, 5, 5, and 5 days, respectively (by stage), to 28 (control), 40, 42, and 44°C.

Temperature (°C)	POD activity (U mg^-1^ protein)			
	Eggs	Larvae	Pupae	Adults
28	15.27 ± 0.52b, A	16.48 ± 0.45a, A	15.78 ± 0.53a, A	14.81 ± 0.87a, A
40	19.32 ± 1.29a, A	15.37 ± 1.14a, BC	12.41 ± 0.55b, C	16.45 ± 0.6a, AB
42	16.43 ± 0.62b, B	25.39 ± 1.2a, A	9.59 ± 0.52c, C	11.59 ± 0.6b, C
44	9.67 ± 0.4c, B	8.48 ± 0.32b, B	8.8 ± 0.28c, B	15.49 ± 1.18a, A

*Values within the same column followed by different lowercase and uppercase letters within the same row are significantly different (Tukey’s HSD test, P < 0.05).*

After long-term phasic thermal stress, the POD activities of *O. communa* female adults increased at 40°C, decreased at 42 and 44°C (*F*_3,8_ = 14.49, *P* = 0.0013); the POD activities of male adults were close to the control at 40 and 42°C, decreased at 44°C (*F*_3,8_ = 4.78, *P* = 0.0341); higher POD activities were also observed in female adults than those of males both at high temperatures and control (**Figure 1D**).

Taken together, antioxidant enzymes (SOD, CAT, and POD) activities were observed for the first time to be sex-dependent – with few exceptions, enzyme activities were significantly higher in female adults than in male adults.

## Discussion

The effects of heat stress on insects depend on the frequency, amplitude, and duration of the stress (Ma et al., 2018), and the sex and developmental stage of the insect (Enriquez and Colinet, 2017). It is reported that the frequency, intensity, and length of heat hot days will increase in the short- and in the long-term (Ma et al., 2018). As an overlapping-generation species with a relatively short generation period (Zhou et al., 2010), any developmental stage or the whole generation of *O. communa* might encounter phasic high-temperature stress. The level of SOD, CAT, and POD activity in *O. communa* adults by the previous exposure of different developmental stages or whole generation to brief high temperatures increased, suggesting the defensive function of these enzymes in abating the adverse effect of ROS generated by the heat stress.

In the present study, we explored the effects of phasic and long-term daily thermal stress on total protein content and enzymatic antioxidant defense systems (SOD, CAT, and POD) of the common ragweed beetle, *O*. *communa*. Our results indicate that these parameters change significantly under different levels of phasic high temperature for 3–5 days, and under long-term high-temperature stress. Protein is reported as one of the major constituents imparting heat tolerance in the red flour beetle, *Tribolium castaneum* (Swetaleena et al., 2013). Overall, the total protein contents in *O*. *communa* adults at high temperatures similar with control (28°C), under long-term thermal stress at 44°C even significantly higher than control (28°C), which suggests that proteins protect *O*. *communa* from heat stress damage. The total protein content was found to increase with thermal stress, and this result is in accordance with a study conducted on *T. castaneum* (Swetaleena et al., 2013). By contrast, a study involving high-temperature treatments of 31°C reported that heat stress decreased protein contents in the termite *Coptotermes formosanus* (Tarver et al., 2012).

Superoxide dismutase is the most important antioxidant enzyme defense system against ROS, which catalyzes the breakdown of superoxide anions and transforms them into hydrogen peroxide and oxygen (Zelko et al., 2002). In the present study, the SOD activity in *O*. *communa* adult males and females increased significantly as a whole under short-term daily phasic high-temperature stress. Under long-term daily phasic high-temperature stress, the SOD activity in *O*. *communa* adult males and females increased at higher temperatures, decreased at 44°C. These results suggest that the activity of SOD might be an adaptive response of different developmental stages *O*. *communa* to overcome high temperature ≤ 44°C induced ROS toxicity, using other superoxide anion scavenging pathways. A previous study indicated that SOD has an important role in reducing the high level of superoxide radicals induced by low or high temperatures (Celino et al., 2011). The increased activities of SOD under high temperature in our results are in accordance with studies of the role of SOD, in the antioxidant responses to thermal stress in the wolf spider, *Xerolycosa nemoralis* (Wilczek et al., 2013), cucumeris mite, *Neoseiulus cucumeris* (Zhang et al., 2014), and *Propylaea japonica* (Zhang et al., 2015a). SOD activity increased significantly at 39°C, and markedly decreased at 41°C after exposure of *P. japonica* adults to heat stress for 1 h compared with control (Zhang et al., 2015a), which indicated that the activity of SOD was induced by moderate heat stress. A significant increase in SOD activities at 36 and 39°C for 1 h compared with control in *Chilo suppressalis* larvae was observed (Cui et al., 2011). SOD activity in the predatory mite *N. cucumeris* was significantly increased compared to the control at 35 and 38°C for 1-3 h (Zhang et al., 2014). Decreased SOD activity could also impair the O_2_-scavenging ability of the cells, thus favoring the accumulation of O_2_ and H_2_O_2_ (Jaleel et al., 2008), as SOD is the first and most important defense against ROS (Feng et al., 2015). Therefore, we hypothesized that SOD also plays a key role in the response of *O*. *communa* to short-term or long-term phasic thermal stress.

Catalase removes H_2_O_2_ only at high cellular concentrations and is inefficient at low concentrations (Yang et al., 2010). In this study, the CAT activities in *O*. *communa* adults were closer to or even higher than the control under short-term daily phasic high-temperature stress. Under long-term daily phasic high-temperature stress, the CAT activities of female *O*. *communa* adults increased at ≤42°C, decreased at 44°C, CAT activities of male *O*. *communa* adults decreased significantly. The present results are in accordance with a study which reported that CAT activity significantly increases at 35–41°C for 1 h in ladybeetle *P. japonica* adults (Zhang et al., 2015a). Increased CAT activity under thermal stress has also been reported in the oriental fruit fly *Bactrocera dorsalis* adults (Jia et al., 2011), and the fifth instar silkworm *Bombyx mori* (Nabizadeh and Kumar, 2011), the rice stem borer *C. suppressalis* larvae (Cui et al., 2011; Lu et al., 2017). These results suggest that CAT provides protection of *O*. *communa* under short-term or long-term phasic thermal stress ≤42°C.

Peroxidase plays an important role in scavenging H_2_O_2_. Under short-term daily phasic high-temperature stress, POD activities increased at 40°C, decreased at 42 or 44°C. Under long-term daily phasic high-temperature stress, POD activities of female *O*. *communa* adults increased at 40°C, decreased at ≥ 42°C, POD activities of male *communa* adults decreased at 44°C. These results are in accordance with POD activity increase in *P. japonica* adults at 41°C for 1 h (Zhang et al., 2015a), and *B. dorsalis* adult at 35–40°C for 3–6 h (Jia et al., 2011). In previous study, POD activity was significantly increased in *N*. *cucumeris* after heat shock for 1–2 h and decreased with the duration of exposure (Zhang et al., 2014). POD had an important role in the antioxidant response to thermal stress in *P. japonica*, and no significant difference in POD activity was found from 35 to 39°C, whereas at 41°C a remarkable increase was observed, compared to the control (Zhang et al., 2015a). We hypothesized that POD also provides protection of *O*. *communa* under short-term or long-term phasic thermal stress ≤ 40°C.

In general, antioxidative enzymes (SOD, CAT, and POD) activity was found to be sex-dependent – it was higher in females than males. Overall, the higher antioxidative enzymes activity of *O*. *communa* females was obtained in the present study, which indicates higher thermal tolerance of female *O*. *communa*. A high body weight, large size, and high survival rate of *O*. *communa* females (Chen et al., 2014; Chen et al. unpublished data) may be closely related to the high antioxidative enzyme activity level of females under heat stress. The stage-specific thermal tolerance is very common in insects (Zhao et al., 2017), the stage-specific antioxidative enzyme activity was reported in *C. suppressalis* (Li et al., 2017), and the stage-specific antioxidative enzyme activity of *O*. *communa* may be related to the sensitivity of stages to heat stress (Chen et al. unpublished data).

In conclusion, thermal stress is one of the factors that can generate oxidative stress products in *O*. *communa*. High-temperature exposures cause oxidative stress at 44°C and changes in antioxidant enzymes (SOD, CAT, and POD) play an important part in reducing oxidative damage in *O. communa* up to 42°C, the increased antioxidant defense systems of SOD, CAT, and POD may be an adaptive response of *O*. *communa* to avoid oxidative stress during exposure to high-temperature stress.

## Author Contributions

ZZ and FW conceived and designed the work. GS and JG contributed to the revision of the manuscript. HC performed the experiments and wrote the manuscript.

## Conflict of Interest Statement

The authors declare that the research was conducted in the absence of any commercial or financial relationships that could be construed as a potential conflict of interest.
